# Scalp acupuncture coupled with speech-language therapy modulates the ventral-dorsal structural brain network in patients with post-stroke anomia: a randomized clinical and DTI neuroimaging trial

**DOI:** 10.1186/s13020-026-01501-1

**Published:** 2026-10-08

**Authors:** Jian Song, Ruiming Nan, Jinglei Ni, Linsong Chai, Lanlan Zhang, Mengxue Wang, Nizhuan Wang, Bingbing Lin, Jia Huang

**Affiliations:** 1 College of Rehabilitation Medicine, Fujian University of Traditional Chinese Medicine, Fuzhou, China; 2 The Affiliated Rehabilitation Hospital, Fujian University of Traditional Chinese Medicine, Fuzhou, China; 3 National-Local Joint Engineering Research Center of Rehabilitation Medicine Technology, Fujian University of Traditional Chinese Medicine, Fuzhou, China; 4 Department of Language Science and Technology, The Hong Kong Polytechnic University, Hung Hom, Kowloon, Hong Kong Special Administrative Region China

**Keywords:** Post-stroke anomia, Scalp acupuncture, Speech-language therapy, Ventral and dorsal structural language network, Diffusion tensor imaging

## Abstract

**Background:**

Scalp acupuncture (SA) is used as adjunctive rehabilitation for post-stroke aphasia, but its effect on naming recovery and structural language-network changes remains unclear. We evaluated SA coupled with speech-language therapy (SLT) in patients with post-stroke anomia and explored diffusion tensor imaging markers of ventral-dorsal structural language networks.

**Methods:**

This randomized controlled trial recruited 85 patients with post-stroke anomia and randomly assigned them to SA or control. Both groups received conventional stroke treatment and SLT; the SA group additionally received scalp acupuncture synchronously with SLT during needle retention. Treatment was delivered once daily, 5 days/week for 4 weeks. The primary outcome was WAB naming total score. Diffusion tensor imaging assessed white matter integrity and topological properties before and after treatment.

**Results:**

Compared to the control group, the SA group showed greater improvements in the WAB naming total score (*P* = 0.009), object naming, sentence completion, responsive speech, BNT spontaneous naming, and SAQOL-39 communication. In the ventral-dorsal structural network, the SA group showed smaller changes in the area under the curve (AUC) of small-worldness properties (*P* = 0.008) and normalized clustering coefficient (*P* = 0.017), and greater changes in clustering coefficient and local efficiency. In the SA group, the change in small-worldness was negatively correlated with the change in the WAB naming total score (r = −0.451, *P* = 0.027). The SA group also showed smaller axial diffusivity (AD) changes in the left uncinate fasciculus (*P* = 0.003, Bonferroni corrected), which correlated negatively with the WAB naming total score change (r = −0.440, *P* = 0.031). Exploratory adjusted regression showed that SA-group assignment was associated with greater naming improvement after adjustment for baseline naming and clinical covariates.

**Conclusions:**

SA coupled with SLT was associated with greater naming recovery in patients with post-stroke anomia. The benefit was accompanied by changes in structural language-network topology and white matter integrity, particularly involving the left UF. These findings support further evaluation of scalp acupuncture as an adjunct to language rehabilitation and suggest structural network plasticity as a relevant imaging correlate.

**Supplementary Information:**

The online version contains supplementary material available at https://doi.org/10.1186/s13020-026-01501-1.

## Introduction

Post-stroke anomia (PSA), the impaired ability to name objects, is a core clinical feature of post-stroke aphasia [1]. Patients with post-stroke aphasia may show naming impairment across objects, pictures, or actions, regardless of aphasia type [2]. Naming requires coordinated perceptual processing, semantic access, and phonological representation. Stroke-related structural damage can disrupt distributed neural networks and white matter tracts that support these processes [3]. Therefore, promoting the recovery of post-stroke anomia has become a central goal in clinical rehabilitation.

Scalp acupuncture (SA) is an acupuncture-based intervention used in traditional Chinese medicine and rehabilitation settings for post-stroke language impairment. Liu Zhizhen et al. have reported significant improvements in overall language function scores for post-stroke aphasia patients treated with SA compared to a control group [4]. However, this “overall language function score” is a composite measure, reflecting contributions from various linguistic domains such as auditory comprehension and repetition. Improvement in a composite language score does not necessarily indicate improvement in naming ability. Because lexical retrieval and naming involve distinct cognitive processes and neural circuits, the clinical efficacy and recovery mechanisms of SA for PSA require direct investigation.

The ventral-dorsal brain network model provides a structural and network basis for language perceptual processing and motor representation [5]. This model divides language processing into the ventral semantic network and the dorsal phonological network. Changes in the integrity of white matter fiber tracts have been associated with language recovery in patients with post-stroke aphasia [6].

PSA is also associated with changes in the white matter fiber tracts [7]. Dysfunction of the white matter fiber tracts within the ventral-dorsal brain network may contribute to naming-related functions. Specifically, lexical-semantic performance has been linked to radial diffusivity in the left arcuate fasciculus, and the integrity of major language pathways has been associated with lexical-semantic and syntactic deficits after left hemisphere strokes [8]. Janina Wilmskoetter et al. found that severe white matter hyperintensities were associated with fewer long-range white matter fibers [9]. Thus, the recovery of naming function may involve integration within the ventral-dorsal network and reorganization of residual networks. Assessing white matter integrity can also reveal abnormalities beyond the lesion site and provide more multidimensional structural information [10].

Recovery of PSA is thought to depend partly on post-stroke neuroplasticity, including changes in cerebral perfusion, metabolism, and structural and functional reorganization of language networks. SA may influence language-related network activity, but direct evidence in naming recovery remains limited. Prior work reported that SA increased local functional activity in the left supramarginal gyrus and Wernicke's area in patients with stroke [11]. Although that study did not directly assess language outcomes, its finding that SA can activate these regions implies a potential for modulating the broader dorsal and ventral pathways that are critical for language processing. Direct evidence is still needed to determine whether SA supports naming recovery through changes in dual-stream structural networks. We therefore investigated the effects of a 4-week intervention combining SA with SLT on naming function and quality of life in patients with PSA. We also explored whether treatment-related recovery was accompanied by changes in ventral and dorsal brain networks quantified using diffusion tensor imaging (DTI) [12, 13].

## Results

### General demographic and clinical characteristics

**Fig. 1:**
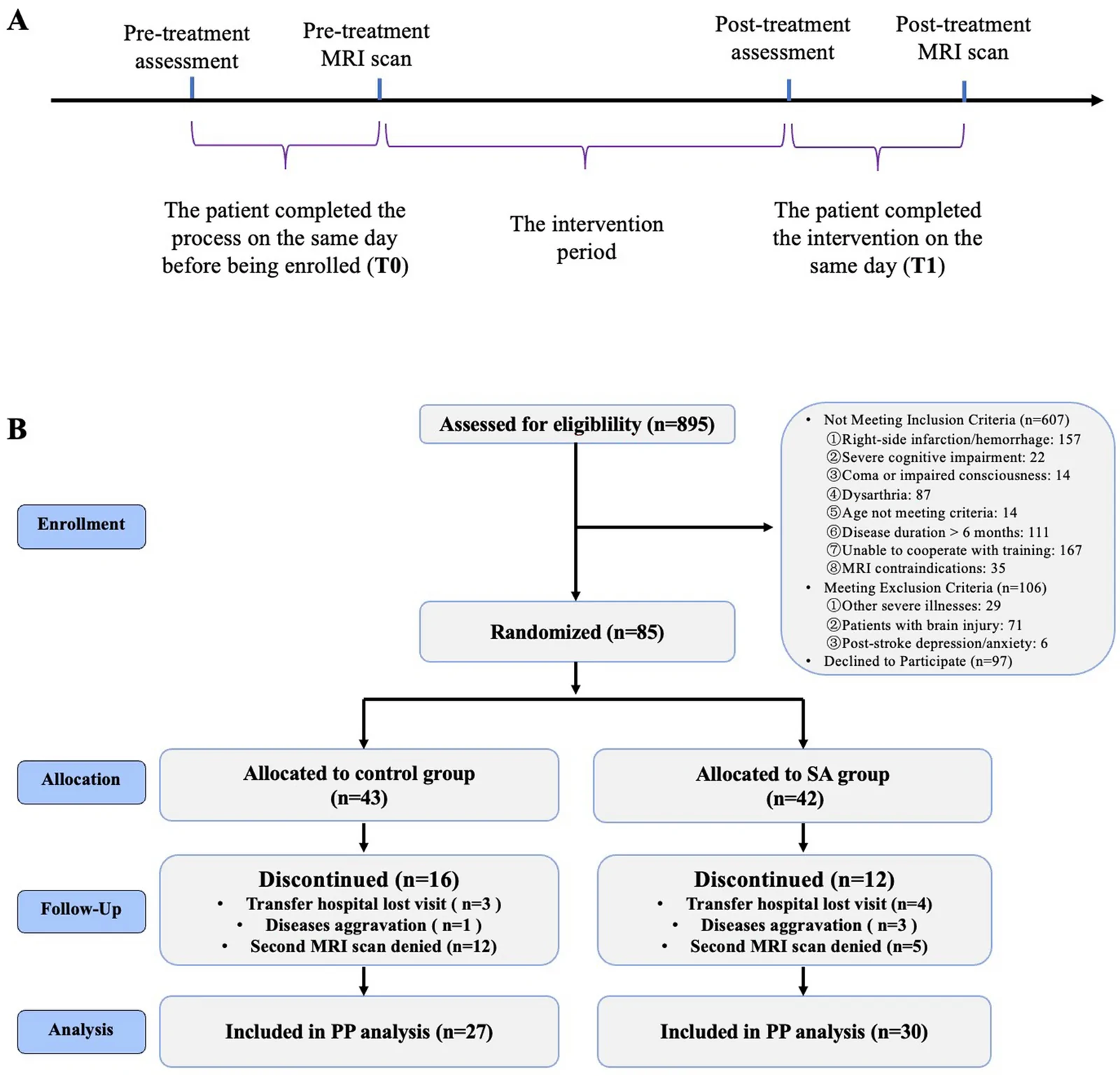
The designed evaluation and intervention process and the implemented flowchart of this study. **A** The assessment and magnetic resonance scanning process of this study. **B** The implemented flowchart of this study. *PP* Per-protocol, *SA* Scalp acupuncture group

There were no statistically significant differences between the SA group and the control group in age, sex, education years, disease duration, stroke type, aphasia type, WAB aphasia quotient (AQ), or BNT spontaneous naming score (*P* > 0.05) (Table 1 and Fig. 1). Baseline neuroimaging comparisons are reported in the Supplementary Materials. Although AD, RD, and MD showed no significant between-group differences across the examined tracts, baseline FA differed in the left IFOF, left ILF, and left UF. For network topology, baseline clustering coefficient and local efficiency also differed between groups.

**Table 1:** Demographic information summary of the SA group and control group

Item	Control group (n = 27)	SA group (n = 30)	t/Z/*x*^2^	*P*
General demographic data				
Age (years)	58.85 ± 13.20	56.60 ± 15.38	t = −0.590	0.558
Sex (male/female)	24/3	22/8	*x*^2^ = 2.208	0.186
Education years (years)	9.00 (6.00,12.00)	9.00 (6.00,15.00)	Z = −0.424	0.696
Disease duration (days)	36.00 (25.00,68.00)	41.50 (29.50,84.50)	Z = −0.919	0.363
Stroke type (ischemic/hemorrhagic)	21/6	21/9	*x*^2^ = 0.443	0.559
Aphasia type (fluent/non-fluent)	6/21	8/22	*x*^2^ = 0.151	0.765
WAB aphasia quotient (AQ)	24.60 (13.50,45.40)	33.70 (19.70,49.20)	Z = −1.191	0.237
WAB naming subtest (WAB)				
Object naming score	6.00 (0.00,14.00)	8.00 (3.00,22.25)	Z = −0.886	0.380
Word fluency score	0.00 (0.00,1.00)	0.00 (0.00,3.25)	Z = −1.051	0.296
Sentence completion score	0.00 (0.00,6.00)	0.00 (0.00,4.50)	Z = −0.045	0.971
Responsive speech score	0.00 (0.00,4.00)	0.00 (0.00,6.00)	Z = −0.848	0.402
WAB naming total score	8.00 (0.00,24.00)	9.00 (4.50,33.00)	Z = −0.917	0.364
Boston Naming Test (BNT)				
Spontaneous naming score	1.00 (0.00,5.00)	3.00 (1.00,6.25)	Z = −0.680	0.501
Selective naming score	4.00 (1.00,9.00)	4.50 (0.75,5.00)	Z = −0.418	0.681
Prompt naming score	3.00 (0.00,5.00)	4.00 (2.00,5.50)	Z = −1.323	0.189
Misnamed score	16.89 ± 8.32	15.47 ± 7.79	t = −0.667	0.508
Stroke and Aphasia Quality of Life Scale (SAQOL-39)				
Physical score	1.82 (1.29,2.71)	2.00 (1.59,2.59)	Z = −1.184	0.240
Communication score	1.88 ± 0.63	1.92 ± 0.66	t = 0.224	0.824
Psychosocial score	2.00 (1.50,2.64)	1.93 (1.50,2.93)	Z = −0.857	0.397
SAQOL-39 total score	71.00 (59.00,101.00)	83.00 (61.00,109.00)	Z = −1.143	0.257

Data are presented as mean ± standard deviation, median (interquartile range), or n, as appropriate. Between-group comparisons were performed using independent-samples t tests, Mann–Whitney U tests, or chi-square tests, as appropriate. The table includes participants with complete clinical and MRI data who were included in the per-protocol imaging analysis. All *P* values are two-sided

### Intergroup comparison of naming function scores and quality of life scores

Compared with the control group, the SA group showed significantly greater improvements (change scores, T1–T0) in the WAB naming total score (*P* = 0.009) and in the object naming (*P* = 0.011), sentence completion (*P* = 0.008), and responsive speech (*P* = 0.013) sub-dimensions. The change in the BNT spontaneous naming score was also significantly higher (T1–T0) in the SA group (*P* = 0.043). In addition, the SA group showed greater improvement (T1–T0) in the communication domain of the SAQOL-39 (*P* = 0.036). No statistically significant between-group differences were observed for the other results (Table 2).

**Table 2:** Intergroup comparison of the differences in naming function and quality of life scores of pre (T0)/post (T1) rehabilitation training

Item	Control group (n = 27)			SA group (n = 30)			t/Z	Effect size	*P*
	T0 (pre)	T1 (post)	T1–T0 (post–pre)	T0 (pre)	T1 (post)	T1–T0 (post–pre)			
WAB naming subtest (WAB)									
Object naming score	6.00 (0.00,14.00)	14.00 (9.00,24.00)	9.00 (4.00,12.00)	8.00 (3.00,22.25)	34.00 (18.50,43.00)	12.50 (8.50,24.50)	Z = −2.537	r = 0.336	**0.011**
Word fluency score	0.00 (0.00,1.00)	2.00 (0.00,8.00)	2.00 (0.00,5.00)	0.00 (0.00,3.25)	5.00 (1.75,8.50)	3.50 (0.00,6.00)	Z = −0.619	r = 0.082	0.541
Sentence completion score	0.00 (0.00,6.00)	2.00 (0.00,6.00)	2.00 (0.00,2.00)	0.00 (0.00,4.50)	6.00 (2.00,10.00)	4.00 (1.50,4.25)	Z = −2.634	r = 0.349	**0.008**
Responsive speech score	0.00 (0.00,4.00)	2.00 (0.00,8.00)	2.00 (0.00,2.00)	0.00 (0.00,6.00)	8.00 (2.00,10.00)	4.00 (0.75,6.25)	Z = −2.475	r = 0.328	**0.013**
WAB naming total score	8.00 (0.00,24.00)	22.00 (12.00,44.00)	14.00 (8.00,21.00)	9.00 (4.50,33.00)	50.00 (30.75,67.75)	21.00 (14.75,41.50)	Z = −2.583	r = 0.342	**0.009**
Boston Naming Test (BNT)									
Spontaneous naming score	1.00 (0.00,5.00)	4.00 (2.00,10.00)	3.00 (1.00,4.00)	3.00 (1.00,6.25)	6.50 (4.00,13.50)	3.50 (2.75,6.00)	Z = −2.022	r = 0.268	**0.043**
Selective naming score	4.00 (1.00,9.00)	5.00 (4.00,8.00)	1.00 (0.00,4.00)	4.50 (0.75,5.00)	5.00 (2.00,8.25)	1.50 (−0.25,3.00)	Z = −0.491	r = 0.065	0.629
Prompt naming score	3.00 (0.00,5.00)	5.00 (0.00,7.00)	1.00 (0.00,3.00)	4.00 (2.00,5.50)	5.50 (2.75,7.25)	1.00 (−1.25,3.25)	Z = −0.097	r = 0.013	0.926
Misnamed score	16.89 ± 8.32	11.00 (5.00,17.00)	−7.00 (−10.00, −3.00)	15.47 ± 7.79	9.50 (4.00,16.00)	−5.00 (−9.25, −2.50)	Z = −0.385	r = 0.051	0.705
Stroke and Aphasia Quality of Life Scale (SAQOL-39)									
Physical score	1.82 (1.29,2.71)	2.88 ± 1.07	0.94 (0.35,1.47)	2.00 (1.59,2.59)	3.35 ± 1.02	1.15 (0.47.1.47)	Z = −1.000	r = 0.132	0.322
Communication score	1.88 ± 0.63	2.80 ± 0.83	0.92 ± 0.63	1.92 ± 0.66	3.20 ± 0.84	1.29 ± 0.66	t = 2.155	g = 0.564	**0.036**
Psychosocial scores	2.00 (1.50,2.64)	2.96 ± 0.91	0.99 ± 0.75	1.93 (1.50,2.93)	3.57 ± 0.93	1.37 ± 0.75	t = 1.945	g = 0.509	0.057
SAQOL-39 total score	71.00 (59.00,101.00)	112.81 ± 34.48	36.30 ± 22.30	83.00 (61.00,109.00)	132.47 ± 34.27	45.13 ± 26.70	t = 1.348	g = 0.353	0.183

Data are presented as mean ± standard deviation or median (interquartile range), as appropriate. Between-group comparisons were performed using change scores from baseline to post-intervention (T1–T0). Independent-samples t tests were used for normally distributed change scores, whereas Mann–Whitney U tests were used for non-normally distributed change scores. Effect sizes are reported as Hedges’ g for independent-samples t tests and as $r\, = \,{{\left| Z \right|} \mathord{\left/ {\vphantom {{\left| Z \right|} {\sqrt N }}} \right. \kern-0pt} {\sqrt N }}$ for Mann–Whitney U tests. Positive effect sizes indicate greater improvement in the scalp acupuncture group. All *P* values are two-sided. Bold *P* values indicate statistically significant between-group differences (*P* < 0.05)

### Intergroup comparison of small-worldness properties and network efficiency in the ventral-dorsal structural brain networks

**Fig. 2:**
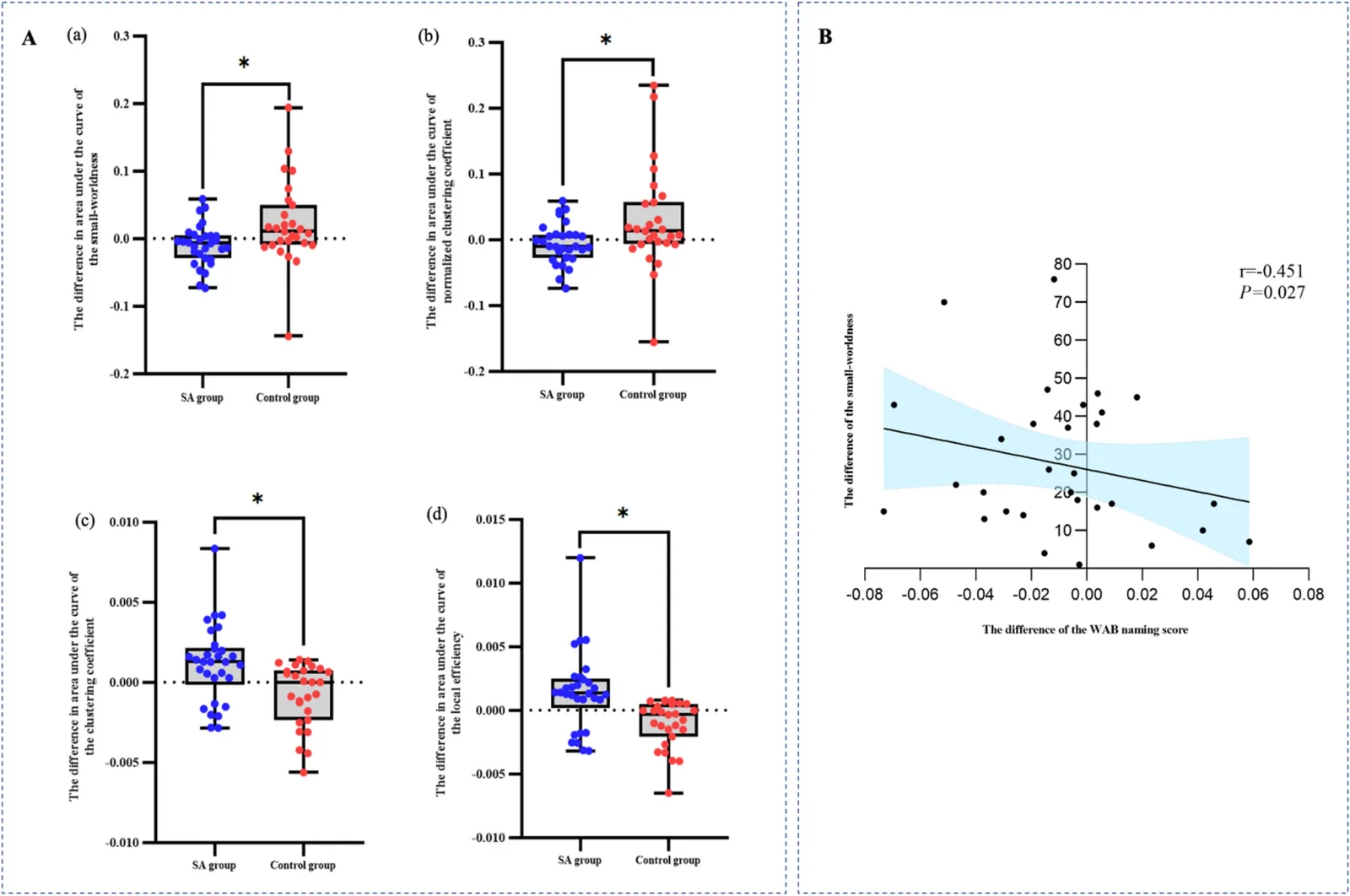
Intergroup comparison of global topological properties in dorsal and ventral brain networks and correlation analysis with naming function scores. **A** (a) Intergroup comparison of the difference (T1–T0) in area under the curve of small-worldness between two groups of subjects. (b) Intergroup comparison of the difference (T1–T0) in area under the curve of normalized clustering coefficient between two groups of subjects. (c) Intergroup comparison of the difference (T1–T0) in area under the curve of clustering coefficient between two groups of subjects. (d) Intergroup comparison of the difference (T1–T0) in area under the curve of local efficiency between two groups of subjects. **B** Correlation between the difference (T1–T0) in area under the curve of small-worldness and the difference (T1–T0) of naming total score of WAB naming subtest in SA group

Analysis of network topological properties revealed that the SA group had a significantly smaller change (T1–T0) in the Area Under the Curve (AUC) for small-worldness (*P* = 0.008) and the normalized clustering coefficient (*P* = 0.017) than the control group. Conversely, the SA group showed a significantly greater change (T1–T0) in the AUC for the clustering coefficient (*P* = 0.011) and local efficiency (*P* < 0.001). After adjustment for age, sex, education years, disease duration, stroke type, and aphasia type, partial correlation analysis revealed a significant negative correlation between the change in the AUC for small-worldness and the change in the WAB naming score within the SA group (r = −0.451, *P* = 0.027). No other statistically significant correlations were observed (Fig. 2).

### The intergroup comparison of AD changes in white matter fiber tracts of the ventral-dorsal structural brain networks

**Fig. 3:**
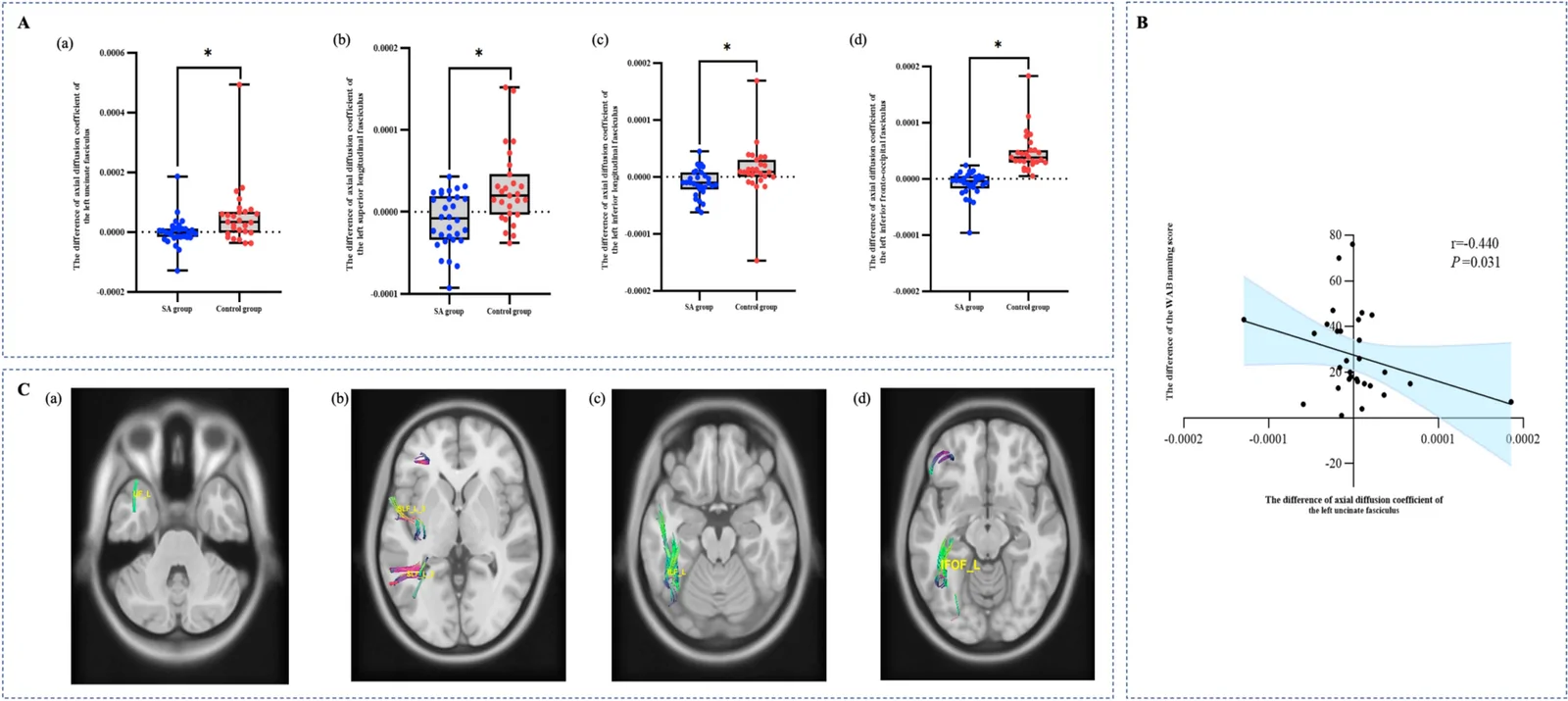
Intergroup comparison of white matter fiber integrity parameters in dorsal and ventral brain networks and correlation analysis with naming function scores. **A** (a) Intergroup comparison of the difference (T1–T0) of axial diffusion coefficient of left uncinate fasciculus (L_UF) between two groups of subjects. (b) Intergroup comparison of the difference (T1–T0) of axial diffusion coefficient of left superior longitudinal fasciculus (L_SLF) between two groups of subjects. (c) Intergroup comparison of the difference (T1–T0) of axial diffusion coefficient of left inferior longitudinal fasciculus (L_ILF) between two groups of subjects. (d) Intergroup comparison of the difference (T1–T0) of axial diffusion coefficient of left inferior fronto-occipital fasciculus (L_IFOF) between two groups of subjects. **B** Correlation between the difference (T1–T0) of axial diffusion coefficient of left uncinate fasciculus (L_UF) and the difference of naming total score of WAB naming subtest in SA group. **C** Representative reconstructions of white matter tracts showing significant between-group differences in axial diffusivity changes: (a) left uncinate fasciculus (L_UF), (b) left superior longitudinal fasciculus (L_SLF), (c) left inferior longitudinal fasciculus (L_ILF), and (d) left inferior fronto-occipital fasciculus (L_IFOF)

Regarding white matter integrity, the SA group exhibited significantly smaller changes (T1–T0) in axial diffusivity (AD) across several key fiber tracts compared to the control group. Specifically, within the ventral semantic network, these differences were observed in the left inferior fronto-occipital fasciculus (IFOF; *P* < 0.001, Bonferroni corrected), the left inferior longitudinal fasciculus (ILF; *P* = 0.001, Bonferroni corrected), and the left uncinate fasciculus (UF; *P* = 0.003, Bonferroni corrected). Similarly, in the dorsal phonological network, the change (T1–T0) in AD in the left superior longitudinal fasciculus (SLF) was also significantly smaller in the SA group (*P* = 0.002, Bonferroni corrected). Furthermore, after adjustment for age, sex, education years, disease duration, stroke type, and aphasia type, partial correlation analysis showed a significant negative correlation between the change in AD in the left UF and the change in the WAB naming total score within the SA group (r = −0.440, *P* = 0.031). No other statistically significant correlations were observed (Fig. 3).

### The intergroup comparison of MD and RD changes in white matter fiber tracts of the ventral-dorsal structural brain networks

The SA group exhibited lower mean diffusivity (MD, T1–T0) change values in the left IFOF (*P* < 0.001, Bonferroni corrected) and the left ILF (*P* = 0.001, Bonferroni corrected) within the ventral semantic network. Additionally, within the dorsal phonological network, the SA group showed lower MD change values in the left SLF (*P* < 0.001, Bonferroni corrected). Furthermore, after adjustment for age, sex, years of education, disease duration, stroke type, and aphasia subtype, the partial correlation analysis revealed no significant association.

**Fig. 4:**
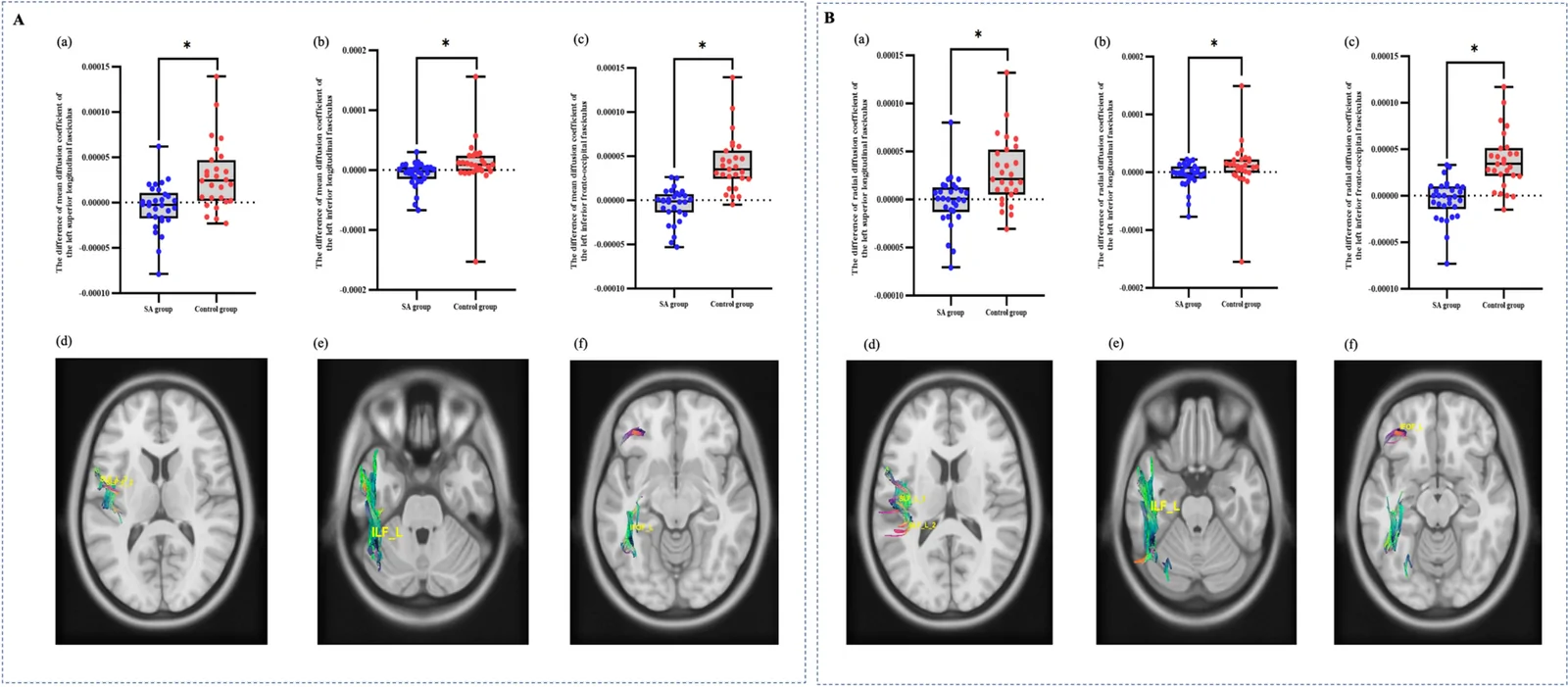
Intergroup comparison of changes in mean diffusivity and radial diffusivity of white matter tracts in the ventral-dorsal structural language network. **A** Mean diffusivity (MD) changes. (a–c) Between-group comparisons of MD changes (T1–T0) in the left superior longitudinal fasciculus (L_SLF), left inferior longitudinal fasciculus (L_ILF), and left inferior fronto-occipital fasciculus (L_IFOF), respectively. (d–f) Representative tract reconstructions of the L_SLF, L_ILF, and L_IFOF, respectively, the tracts with significant between-group differences in MD changes. **B** Radial diffusivity (RD) changes. (a–c) Between-group comparisons of RD changes (T1–T0) in the L_SLF, L_ILF, and L_IFOF, respectively. (d–f) Representative tract reconstructions of the L_SLF, L_ILF, and L_IFOF, respectively, the tracts with significant between-group differences in RD changes

Similarly, the SA group demonstrated lower radial diffusivity (RD, T1–T0) in the left IFOF (*P* < 0.001, Bonferroni corrected) and the left ILF (*P* = 0.006, Bonferroni corrected) within the ventral semantic network. Furthermore, within the dorsal phonological network, the SA group showed lower RD (T1–T0) in the left SLF (*P* < 0.001, Bonferroni corrected). Furthermore, after adjustment for age, sex, years of education, disease duration, stroke type, and aphasia subtype, the partial correlation analysis revealed no significant association (Fig. 4).

### Exploratory regression analysis of imaging changes associated with naming recovery

In the exploratory hierarchical regression analysis, Model 1, including treatment assignment, baseline WAB naming total score, and clinical covariates, explained 26.9% of the variance in naming improvement. Assignment to the SA group remained associated with greater improvement in WAB naming total score (B = 12.931, standardized β = 0.378, *P* = 0.005). After theoretically selected imaging changes, including left IFOF AD, left UF AD, and small-worldness, were added to the model, the explained variance increased from 0.269 to 0.292, but this increment was not statistically significant (ΔR^2^ = 0.023, F-change = 0.487, *P* = 0.693). None of the imaging changes was independently associated with naming improvement after adjustment. Sensitivity analyses excluding influential observations and BCa bootstrap confidence intervals yielded consistent results.

## Discussion

### Effects of SA coupled with SLT on network topology properties and white matter integrity metrics

Compared with the control group, the SA group showed distinct between-group differences in changes in topological properties of the ventral-dorsal structural language network. These included smaller changes in small-worldness and normalized clustering coefficient, together with greater changes in clustering coefficient and local efficiency. Concerning white matter tract integrity within these networks, the SA group exhibited significant alterations in the AD of the left IFOF, left ILF, left SLF, and left UF. Additionally, significant alterations were observed in the MD of the left IFOF, left ILF, and left SLF, as well as in the RD of these fasciculi. Together, these findings indicate that the clinical benefit of SA coupled with SLT was accompanied by changes in ventral-dorsal structural network topology and white matter tract integrity. These imaging changes provide a neurostructural perspective for understanding the adjunctive therapeutic effect of SA, although their predictive and mechanistic implications require further validation.

Compared with the control group, the SA group demonstrated a significantly greater improvement in WAB naming scores, indicating a measurable enhancement in lexical retrieval ability. Although a formal minimal clinically important difference has not been established for the WAB naming subtest, the magnitude of change, together with concurrent improvement in the communication domain of the SAQOL-39, suggests that the additional benefit associated with SA coupled with SLT was not limited to test performance but was accompanied by functional communicative gains. These behavioral findings support the clinical relevance of the structural network alterations observed following treatment.

### Ventral and dorsal network plasticity following SA coupled with SLT and its association with naming recovery

Patients with PSA showed significant improvement in naming function scores after SA coupled with SLT treatment. The imaging findings suggest that recovery in the SA group may involve plasticity in both the ventral and dorsal streams. Within the ventral semantic network, which plays a crucial role in picture naming and semantic control, the UF is a key long-range fiber bundle connecting the anterior temporal lobe with the orbitofrontal cortex, making it critical for lexical retrieval [14, 15].

In our study, we observed that the change in AD of the left UF was significantly negatively correlated with the improvement in WAB naming scores. Research has shown that in patients with PSA, the AD value of the left UF pathologically increases, which may be related to lesion damage [16]. However, our research results reveal that after intervention, the AD of the left UF decreases, indicating a structural remodeling phenomenon, and is associated with the improvement of naming scores [17, 18]. Recent studies have also found that the uncinate fasciculus is closely related to short-term speech memory [19]. Broadly, the ventral semantic network plays a key role in mapping phonetic representations of sounds to distributed conceptual representations [20]. Specifically, the left UF specifically participates in “top-down” control during semantic processing, particularly in word retrieval and semantic interference control [21].

However, behavioral recovery in aphasia is rarely reliant on a single pathway. Alongside the ventral stream, our findings indicate significant microstructural alterations (AD, MD, RD) in the left SLF. As a primary tract of the dorsal stream, the SLF is heavily implicated in phonological processing and articulatory networks; its preserved microstructural integrity is essential for mapping phonemic sequences onto motor plans during word production [22]. The concurrent alterations in the left IFOF, ILF, UF, and SLF suggest that SA coupled with SLT promotes a dual-stream remodeling process. Within the ventral stream, the IFOF and ILF contribute to semantic access by linking posterior perceptual regions with anterior temporal and frontal areas [23], while the UF supports lexico-semantic retrieval and top-down control during word selection [24]. The parallel mitigation of diffusivity changes across these ventral tracts reflects a shared restorative process that strengthens the semantic foundation for naming.

Simultaneously, the dorsal stream SLF remodeling enhances phonological encoding and articulatory programming, ensuring that retrieved semantic information can be translated into accurate spoken output. Rather than acting solely on semantic processing, this combined therapy therefore supports naming recovery by facilitating semantic retrieval via the ventral pathways and phonological representation via the dorsal pathways, thereby restoring the coordinated neural dynamics necessary for successful lexical production [25].

### Regulatory effects of SA coupled with SLT on topological properties of the ventral-dorsal network

In addition to localized tract improvements, this study found that subjects in the SA group showed a decrease in both small-worldness and the normalized clustering coefficient. After a stroke, the topological properties of brain structural networks are affected [26]. One study showed that stroke patients exhibit abnormal small-world characteristics and network properties compared to healthy individuals [27], manifested as enhanced small-worldness [28]. Yifei Zhu et al. found that, compared to healthy controls, stroke patients' brain networks exhibit altered topological organization, which disrupts the balance between local processing efficiency and overall information transmission efficiency [29]. This specifically manifests as a lower shortest path length and higher global efficiency.

Additionally, the topological properties of the network dynamically change over time following a stroke. Small-world properties represent a balance between information integration and segregation within the network, while the normalized clustering coefficient reflects the isolation strength of brain information cluster networks [30]. Therefore, this study speculates that the ability for information integration and segregation in the brain networks of patients with post-stroke anomia is disrupted, resulting in increased information isolation and reduced information flow. SA coupled with SLT may therefore reduce information isolation in the ventral-dorsal structural brain networks, enhance the efficiency of information transmission in small-world networks, and thus promote the recovery of naming function.

Compared to the control group, subjects in the SA group exhibited an increased clustering coefficient in the ventral-dorsal structural brain networks. The clustering coefficient is a measure of functional segregation, representing the ability of tightly interconnected brain regions to process specialized information [30]. Small-world networks, characterized by a short characteristic path length and a high clustering coefficient, ensure efficient information differentiation and integration. Additionally, local efficiency is related to the clustering coefficient; an increase in either metric represents enhanced local network information processing efficiency.

Moreover, subjects in the SA group showed an increased local efficiency in the ventral-dorsal structural brain networks. Local efficiency measures the ability of local regions within a network to transmit information [30], and also reflects the network's resilience to random attacks and fault tolerance. A decrease in local efficiency can impair language information exchange and processing in post-stroke aphasia patients. Xiaoyun Chen et al. [31] reported that aphasia patients have reduced local efficiency. Furthermore, they found this metric is positively correlated with naming and auditory comprehension abilities, suggesting that the capability for information exchange and regional information processing is disrupted. Other studies have found that higher local efficiency indicates stronger small-world characteristics, suggesting a shift towards an organization that enhances information transmission efficiency.

Thus, this study speculates that SA coupled with SLT may regulate the structural plasticity in post-stroke anomia [32]. This regulation may involve dynamically adjusting the functional segregation and integration of abnormal brain networks [33], restoring balance in abnormal brain function states [34]. Ultimately, these changes could promote the recovery of language functions in the ventral-dorsal brain networks, improving language abilities in patients with anomia.

### Exploratory regression analysis and interpretation of imaging markers

The exploratory hierarchical regression analysis further showed that treatment assignment remained associated with naming improvement after adjustment for baseline naming performance and clinical covariates. However, the addition of theoretically selected imaging changes did not significantly improve model performance. Therefore, the observed changes in left IFOF AD, left UF AD, and small-worldness should be interpreted as treatment-related imaging correlates rather than validated predictors. Larger studies incorporating pretreatment imaging assessments and independent validation are needed to determine whether structural network measures are associated with individual differences in treatment response [35, 36].

### Limitations

This study has several limitations. First, the follow-up period was limited to the immediate post-intervention assessment, and the long-term durability of the therapeutic effect of SA coupled with SLT remains unknown. Second, although randomization was performed and baseline clinical characteristics were generally balanced, the control group had slightly lower baseline language scores. Third, the neuroimaging analyses were based on a modest per-protocol sample with complete MRI data, and some baseline imaging metrics differed between groups; therefore, treatment-related imaging changes should be interpreted cautiously. Fourth, the regression analyses were exploratory. Although adjusted hierarchical regression, influence analysis, and bootstrap sensitivity analyses were performed, the imaging measures did not show significant incremental explanatory value beyond treatment assignment and clinical covariates. Fifth, the control condition did not include sham acupuncture, and participants and therapists could not be blinded to the SA intervention. Future studies with larger samples, longer follow-up, theory-informed imaging measures, and independent validation are needed.

## Conclusions

This randomized controlled trial found that scalp acupuncture coupled with speech-language therapy was associated with greater improvement in naming function in patients with post-stroke anomia. The clinical benefit was accompanied by treatment-related changes in the ventral-dorsal structural network topology and white matter integrity, particularly involving the left UF and left IFOF. These findings support further investigation of scalp acupuncture as an adjunctive approach to language rehabilitation and suggest that structural network plasticity may be a relevant imaging correlate. Larger studies with longer follow-up are needed to validate these imaging markers.

## Methods

### Study design

The study used a randomized controlled trial design. The trial was approved by the Ethics Committee of the Affiliated Rehabilitation Hospital of Fujian University of Traditional Chinese Medicine (No. 2021KY-001-01) and registered at the Chinese Clinical Trial Registry (No. ChiCTR2200062146).

### Participants

From July 2022 to July 2023, 85 patients with post-stroke anomia were recruited at the Rehabilitation Hospital Affiliated with Fujian University of Traditional Chinese Medicine.

Inclusion criteria: (1) Diagnosis of cerebral ischemia or hemorrhage in the left hemisphere, confirmed by computed tomography (CT) or magnetic resonance imaging (MRI). (2) Western Aphasia Battery (WAB) aphasia quotient (AQ) < 93.8 score, indicating post-stroke aphasia. (3) Boston Naming Test (BNT-30) spontaneous naming examination < 20 score, indicating anomia [37]. (4) Age between 45 and 85 years. (5) First stroke, with disease duration between 2 weeks and 6 months (24 weeks) [38–40]. (6) Premorbid right-handedness and no contraindications to MRI scanning. (7) Provided written informed consent and were able to understand and cooperate with the assessments and treatments.

Exclusion criteria: (1) Aphasia secondary to other diseases (e.g., brain tumor, traumatic brain injury). (2) Speech difficulties caused by dysarthria. (3) Severe auditory, visual, or cognitive impairments that precluded cooperation with language assessments or MRI scanning.

All participants underwent a baseline assessment and an initial MRI scan on the same day, prior to the commencement of the 4-week intervention. Following the treatment period, a post-intervention assessment and a second MRI scan were conducted, also on the same day that the intervention concluded (Fig. 1).

### Randomization and blinding

Eligible participants were randomly allocated to either the SA group or the control group at a 1:1 ratio. Outcome assessors and statisticians were blinded to group assignments. Allocation concealment was achieved using the sealed opaque envelope method. For details, please refer to the supplementary materials.

### Interventions

#### Control group treatment protocol

The control group received conventional stroke treatment, which primarily included conventional medical treatment, health education, and SLT. Participants received speech rehabilitation training for 30 min per session, once daily, 5 days a week, lasting a period of 4 weeks [41]. For details, please refer to the supplementary materials.

#### Scalp acupuncture group treatment protocol

Patients in the scalp acupuncture group received a combination of scalp acupuncture therapy and speech-language therapy. The treatment was administered by a professional acupuncture therapist using disposable, sterile Hua Tuo brand acupuncture needles (specification: 0.25 × 40 mm). For the treatment, the therapist targeted acupoints on the left side of the patient's head: the lower 2/5 of the Anterior Oblique Line of the Temporal region and the Anterior Temporal Line. During the procedure, the needle was inserted quickly at a 15–30° angle to the scalp to a depth of approximately 15–25 mm, ensuring it reached the subgaleal layer. Immediately after insertion, a rapid twirling manipulation (approximately 200 times/minute) was applied for 1 min to elicit the “Deqi” sensation. After Deqi was achieved, the needles were retained for 30 min.

During this retention period, the needles were manually stimulated by twisting every 10 min, for a total of two stimulations, before removal. Speech rehabilitation training began immediately after “Deqi” was achieved and was conducted concurrently with needle retention. The entire treatment regimen was administered once daily, 5 days a week, for a total duration of 4 weeks.

### Outcome measures

#### Primary outcome measure

The primary outcome was the total score of the naming function subtest of the Western Aphasia Battery (WAB). This subtest comprises four components: object naming, word fluency, sentence completion, and responsive speech. The maximum score is 100, with higher scores reflecting superior naming performance.

#### Secondary outcome measures

1. The Boston Naming Test (BNT) consists of 30 drawings that are graded according to familiarity, ranging from frequently occurring familiar objects to less frequently occurring objects.
2. The Stroke and Aphasia Quality of Life Scale (SAQOL-39) includes three domains: physical, communication, and psychosocial. This scale consists of 39 items rated on a 5-point Likert scale (1–5), with higher scores indicating a better quality of life.
3. Changes in the WAB Aphasia Quotient (WAB AQ), which reflects aphasia severity, were also monitored. The AQ score ranges 0 to 100, and an AQ score < 93.8 is diagnostic for aphasia.

### DTI data preprocessing and analysis

Preprocessing was performed using PANDA [42] software, based on the MATLAB 2018b (The MathWorks, Inc) platform. The raw MRI data were converted into NIfTI format files. Correction for eddy currents and head motion was applied to the DTI data. The surface neighbor method was used to calculate the correlation between a given voxel and the diffusion metrics of its 7 adjacent voxels. The Fractional Anisotropy (FA) map for each participant was normalized to the Montreal Neurological Institute (MNI) space. The resulting parameter maps were resampled to a voxel size of 2 × 2 × 2 mm^3^ and smoothed with a 6 mm full-width at half-maximum (FWHM) Gaussian kernel to reduce noise and registration errors [42]. For details of the analyses of white matter tract integrity and global topological properties of the ventral-dorsal structural brain network, please see the Supplementary Materials.

### Exploratory hierarchical regression and sensitivity analysis of imaging changes associated with naming recovery

An exploratory hierarchical linear regression analysis was performed to examine whether treatment-related neuroimaging changes provided additional explanatory value for naming recovery. The change in WAB naming total score from baseline to post-intervention was specified as the dependent variable. Model 1 included treatment assignment, baseline WAB naming total score, age, sex, education years, disease duration, stroke type, and aphasia type. Model 2 additionally included three theory-informed imaging change measures: left IFOF axial diffusivity change, left UF axial diffusivity change, and small-worldness change. Incremental explanatory value was assessed using the change in R^2^ and the F-change test. Model diagnostics included assessment of collinearity, residual distribution, studentized deleted residuals, Cook’s distance, and leverage values. Sensitivity analyses were performed by excluding influential observations and by calculating bias-corrected and accelerated bootstrap 95% confidence intervals. Details are provided in the Supplementary Materials.

### Statistical analysis

Statistical analysis was performed on the Per-Protocol (PP) population, which included all randomized subjects who completed the full course of treatment as predefined in the protocol and had no major protocol deviations. SPSS 26.0 software was used for statistical analysis. Statistical tests were performed using two-sided tests. When *P* < 0.05, the inter-group comparison was statistically significant.

For continuous data, changes from baseline (post-treatment value minus pre-treatment value, T1–T0) were calculated. Between-group comparisons of these changes were performed using the independent samples t-test for normally distributed data, or the Mann–Whitney U test for non-normally distributed data. Within-group comparisons were performed using the paired t-test for normally distributed data, or the Wilcoxon signed-rank test for non-normally distributed data. For categorical data, the chi-square test was used for between-group comparisons as appropriate. For imaging metrics with significant baseline imbalance, between-group comparisons of post-intervention changes were further examined using analysis of covariance (ANCOVA), with the corresponding baseline values as a covariate.

## Supplementary Information

Supplementary material 1.

## Data Availability

The data presented in this study can be made available upon request from the corresponding authors. However, due to privacy and ethical concerns, the data cannot be publicly disclosed.

## Abbreviations

AD: Axial diffusivity
AQ: Aphasia quotient
AUC: Area under the curve
BNT: Boston Naming Test
CT: Computed tomography
DTI: Diffusion tensor imaging
FA: Fractional anisotropy
FACT: Fiber assignment by continuous tracking
IFOF: Inferior frontal-occipital fasciculus
ILF: Inferior longitudinal fasciculus
MD: Mean diffusivity
MRI: Magnetic resonance imaging
PSA: Post-stroke anomia
RD: Radial diffusivity
SA: Scalp acupuncture
SAQOL-39: Stroke and Aphasia Quality of Life Scale
SLF: Superior longitudinal fasciculus
UF: Uncinate fasciculus
WAB: Western Aphasia Battery
