# Supplementary material for: Scalp acupuncture coupled with speech-language therapy modulates the ventral-dorsal structural brain network in patients with post-stroke anomia: a randomized clinical and DTI neuroimaging trial
Source: Chin Med. 2026 Oct 8;21:226. doi: 10.1186/s13020-026-01501-1 (PMC13647535; doi:10.1186/s13020-026-01501-1)
Supplement: Supplementary file 1 [file 13020_2026_1501_MOESM1_ESM.docx]

# Scalp acupuncture coupled with speech-language therapy modulates the ventral-dorsal structural brain network in patients with post-stroke anomia: A randomized clinical and DTI neuroimaging trial

# Supplementary materials

# Jian Song^1,2,#^, Ruiming Nan^1,#^, Jinglei Ni^1,#^, Linsong Chai^1^, Lanlan Zhang^1^,

# Mengxue Wang^1^, Nizhuan Wang^4^, Bingbing Lin^1,3,*^, Jia Huang^1,3,*^

^1^College of Rehabilitation Medicine, Fujian University of Traditional Chinese Medicine, Fuzhou, China.

^2^The Affiliated Rehabilitation Hospital, Fujian University of Traditional Chinese Medicine, Fuzhou, China.

^3^National-Local Joint Engineering Research Center of Rehabilitation Medicine Technology, Fujian University of Traditional Chinese Medicine, Fuzhou, China.

^4^Department of Language Science and Technology, The Hong Kong Polytechnic University, Hung Hom, Kowloon, Hong Kong Special Administrative Region, China

^#^These authors contributed equally to this work.

^*^Co-corresponding authors.

^*^Correspondence:

Jia Huang, E-mail: [jasmine1874@163.com](mailto:jasmine1874@163.com)

Bingbing Lin, E-mail: [lbbandsw@hotmail.com](mailto:lbbandsw@hotmail.com)

**Methods**

**1. Randomization**

A random number range was predefined to allocate participants into two groups. Subjects were assigned to group A or group B according to the random number table. SPSS 26.0 statistical software was used to generate a random number table. The random grouping results generated by the program were printed out and sealed. The random grouping sequence was concealed using an opaque sealed envelope method. Subjects with post-stroke aphasia and naming dysfunction who met the inclusion and exclusion criteria were randomly assigned to the scalp acupuncture group or the control group in a 1:1 ratio.

**2. Blinding**

This study was blinded to the evaluators and statisticians. Blinding was maintained by an independent researcher who was not involved in the treatment, evaluation, and statistical process. A two-level blinding procedure was adopted. With the primary blind using “A or B” to represent the group to which the subject was assigned. The secondary blind used “A or B” to represent the treatment measure.

**3. Sample size**

This study used the total naming score of the Western Aphasia Battery Scale as the primary outcome measure. According to previous literature reports, the WAB naming total score in the scalp acupuncture group increased from 54.81 ± 28.87 before the intervention to 70.74 ± 25.84 after the intervention. The effect value calculated using G*Power software was 0.397. If a test power of 0.9 is to be obtained, the sample size required for each group is 35 participants, for a total of 70 participants. Considering the dropout rate of 20%, the required total sample size was 85 participants.

**4. Interventions**

**4.1 Control group treatment protocol**

1) Conventional medical treatment, such as addressing the underlying cause of the stroke and managing blood pressure. 2) Health education, with rehabilitation doctors providing health education to patients once a week. 3) Speech-language therapy administered by licensed therapists, primarily targeting naming and lexical retrieval functions.

All participants in both groups received speech-language therapy (SLT) delivered by licensed speech therapists. Therapy duration, frequency, therapist allocation, and total treatment exposure were identical between groups. Because all participants met the inclusion criteria for post-stroke anomia, lexical retrieval impairment was defined a priori as the primary therapeutic target. Naming rehabilitation therefore constituted the dominant component of each treatment session.

The SLT program included language imitation, word-picture matching, picture naming training, repetition exercises, and structured semantic and phonological cueing techniques. While task difficulty was adjusted according to individual performance, the therapeutic objectives and proportional emphasis on naming were standardized across participants. The SLT protocol was standardized across groups to minimize between-group variability in rehabilitation exposure and to isolate the additive therapeutic effect of scalp acupuncture.

The core naming-focused module included: ①Confrontation picture naming tasks using high- and low-frequency items. ②Semantic feature analysis and structured semantic cueing. ③Phonological cueing and phonemic initiation strategies. ④Repetition-facilitated lexical retrieval. ⑤Hierarchically graded cue withdrawal to promote independent word retrieval.

These naming interventions were implemented systematically across all participants and consistently maintained as the central therapeutic focus across sessions.

5. **DTI scanning parameters**

MRI scans were collected using a Siemens Prisma 3.0 T MRI scanner and a 64-channel combined head and neck coil. During the scanning process, subjects were instructed to remain awake. This study utilized a multimodal scanning method to collect T1 structural images and diffusion tensor imaging (DTI) data. The specific scanning parameters are as follows:

1) T1 structural image: sagittal plane scan, repetition time (TR) = 2000 ms, echo time (TE) = 1.73 ms, flip angle = 15 degrees, slice thickness = 1 mm, field of view (FOV) = 240 mm×240 mm, matrix = 256×256, number of slices = 160;

2) DTI imaging: repetition time (TR) = 5000 ms, echo time (TE) = 69 ms, flip angle = 90 degrees, slice thickness = 3.5mm, field of view (FOV) = 230 mm×230 mm, matrix = 64×64, voxel size = 1.8×1.8×3.5 mm³, number of slices = 35.

**6. Analysis of white matter tract integrity in ventral-dorsal structural brain networks**

In this study, eight white matter fiber bundles of interest, closely related to the “ventral-dorsal brain network”, were extracted from the JHU_ICBM atlas ^[1-3]^. These included the bilateral inferior fronto-occipital fasciculus, bilateral inferior longitudinal fasciculus, bilateral superior longitudinal fasciculus, and bilateral uncinate fasciculus. The fractional anisotropy (FA), mean diffusivity (MD), axial diffusivity (AD), and radial diffusivity (RD) of the white matter fiber bundles in the regions of interest for each subject were calculated, and post hoc tests were performed.

Change scores were calculated for each participant as post-treatment minus pre-treatment (T1–T0), and the change scores were then compared between groups. For normally distributed data, a independent-samples t test was used, while Mann–Whitney U test were applied for non-normal data. As this study focused on eight white matter fiber bundles in the ventral-dorsal structural brain network, Bonferroni correction was applied for multiple comparisons, setting the significance threshold at *P* < 0.00625 (0.05/8).

**7. Fibers of interest in the ventral-dorsal structural brain network**

| **Supplementary Table S1** Fibers of interest in the ventral-dorsal structural brain network | | |
| --- | --- | --- |
| Num | Network | White matter fibers |
| 1 | Ventral semantic network | Left Inferior Fronto-occipital Fasciculus (L_IFOF) |
| 2 |  | Right Inferior Fronto-occipital Fasciculus (R_IFOF) |
| 3 |  | Left Inferior Longitudinal Fasciculus (L_ILF) |
| 4 |  | Right Inferior Longitudinal Fasciculus (R_ILF) |
| 5 |  | Left Uncinate Fasciculus (L_UF) |
| 6 |  | Right Uncinate Fasciculus (R_UF) |
| 7 | Dorsal phonological network | Left Superior Longitudinal Fasciculus (L_SLF) |
| 8 |  | Right Superior Longitudinal Fasciculus (R_SLF) |

**8.Construction and Topological Analysis of Dorsal and Ventral Brain Network Connectivity Matrix**

In this study, we constructed dorsal and ventral structural brain networks using the deterministic fiber tracking method. We utilized the Fiber Assignment by Continuous Tracking (FACT) algorithm to build the connectivity matrix of these networks. Tracking was terminated if the angular threshold between two consecutive movements was between 0.2-1 or if the angle exceeded 45° ^[4, 5]^. This study was inspired by the work of Gregory Hickok and David Poeppel on “dorsal and ventral brain networks” ^[1, 2]^. The network nodes were defined based on the JHU brain atlas from Johns Hopkins University^[6]^, covering a total of 20 brain regions.

Topological properties were analyzed using GRETNA ^[7]^ on the MATLAB R2018b platform (The MathWorks, Inc.). We calculated the area under the curve (AUC) of the global topological properties of the dorsal and ventral brain networks in their binary network states, with a sparsity threshold ranging from 0.05 to 0.4 ^[8]^, incremented by 0.05.

The study primarily focused on analyzing the global topological properties of the dorsal and ventral brain networks. These global properties included small-world properties (small-worldness, normalized clustering coefficient, normalized characteristic path length, clustering coefficient, and characteristic path length) and network efficiency (global efficiency and local efficiency).

In our analysis, we performed statistical assessments and post-hoc tests on the AUC of the small-world properties and network efficiency metrics obtained through graph theory analyses. For each participant, change scores were calculated as T1–T0, and between-group comparisons of these change scores were performed. Differences following a normal distribution were assessed using an independent-samples t-test, while non-normally distributed data were analyzed with non-parametric rank-sum tests. *P* ＜ 0.05 was considered statistically significant for inter-group comparisons. In the partial correlation analysis, we controlled for factors such as age, gender, education level, stroke duration, stroke type, and aphasia type, and examined the partial correlation with the main outcome measure, the difference in total naming scores on the Western Aphasia Battery.

| **Supplementary Table S2** Distribution of brain regions in the ventral-dorsal structural brain network | |
| --- | --- |
| Dorsal and ventral brain networks  (20 brain regions) | Brain region |
| Ventral semantics network  (9 brain regions) | Left Angular Gyrus, Left Superior Temporal Gyrus, Left Temporal Pole of the Superior Temporal Gyrus, Left Middle Temporal Gyrus, Left Temporal Pole of the Middle Temporal Gyrus, Left Inferior Temporal Gyrus, Left Posterior of Superior Temporal Gyrus, Left Posterior of Middle Temporal Gyrus, Left Middle Occipital Gyrus. |
| Dorsal phonological network  (7 brain regions) | Left Posterior of Middle Frontal Gyrus, Left Opercular Part of the Inferior Frontal Gyrus, Left Triangular Part of the Inferior Frontal Gyrus, Left Postcentral Gyrus, Left Precentral Gyrus, Left Putamen, Left Globus Pallidus. |
| Regions shared by the ventral semantic and dorsal phonological networks  (4 brain regions) | Left Orbital Part of the Inferior Frontal Gyrus, Left Supramarginal Gyrus, Left Insular Cortex, Left Posterior of Insular Cortex |

**9. Exploratory hierarchical regression and sensitivity analyses**

Exploratory hierarchical linear regression analyses were performed among participants with complete clinical and neuroimaging data. Change in WAB naming total score from baseline to post-intervention (T1–T0) was specified as the dependent variable. Model 1 included treatment assignment, baseline WAB naming total score, age, sex, education years, disease duration, stroke type, and aphasia type. Model 2 additionally included changes in axial diffusivity of the left inferior fronto-occipital fasciculus, axial diffusivity of the left uncinate fasciculus, and small-worldness.

Incremental explanatory value of the imaging measures was evaluated using the change in R² and the F-change test. Collinearity was assessed using variance inflation factors and condition indices. Model diagnostics included evaluation of residual distributions, studentized deleted residuals, Cook’s distance, and leverage values. A sensitivity analysis excluded observations with Cook’s distance greater than 4/n. Bias-corrected and accelerated bootstrap 95% confidence intervals for unstandardized regression coefficients were obtained from 5,000 resamples.

**Results**

**1.Comparison of language function scores before and after treatment in the control group**

Following the intervention, the control group demonstrated significant improvements across all measured language-related outcomes. Within-group analyses showed that, compared with baseline (T0), post-intervention scores on the WAB were significantly increased for object naming, word fluency, sentence completion, responsive speech, and total naming (all *P* < 0.05).

Similarly, relative to baseline (T0), post-intervention scores on the BNT, including spontaneous naming, selective naming, and prompt naming, were significantly higher (all *P* < 0.05). Correspondingly, BNT misnamed scores were significantly reduced after the intervention (*P* < 0.05), indicating improved naming accuracy.

Compared with baseline (T0), scores on the SAQOL-39, including the physical, communication, and psychosocial scores, as well as the SAQOL-39 total score, were all significantly improved after the intervention (all *P* < 0.05) (Table S3).

| **Supplementary Table S3 Within-group comparison of language function in the control group pre- and post-intervention** | | | | |
| --- | --- | --- | --- | --- |
| Item | Control group (n = 27) | | t/Z | *P* |
|  | T0 (pre) | T1 (post) |  |  |
| **WAB Naming Subtest (WAB)** | | | | |
| Object naming score | 6.00  (0.00,14.00) | 14.00  (9.00,24.00) | -4.347 | **<0.001** |
| Word fluency score | 0.00  (0.00,1.00) | 2.00  (0.01,8.00) | -3.632 | **<0.001** |
| Sentence completion score | 0.00  (0.00,6.00) | 2.00  (0.01,6.00) | -2.801 | **0.003** |
| Responsive speech score | 0.00  (0.00,4.00) | 2.00  (0.01,8.00) | -3.653 | **<0.001** |
| WAB naming total score | 8.00  (0.00,24.00) | 22.00  (12.00,44.00) | -4.338 | **<0.001** |
| **Boston Naming Test (BNT)** | | | | |
| Spontaneous naming score | 1.00  (0.00,5.00) | 4.00  (2.00,10.00) | -4.061 | **<0.001** |
| Selective naming score | 4.00  (1.00,9.00) | 5.00  (4.00,8.00) | -2.109 | **0.034** |
| Prompt naming score | 3.00  (0.00,5.00) | 5.00  (0.01,7.00) | -2.136 | **0.033** |
| Misnamed score | 16.89±8.32 | 11.00  (5.00,17.00) | -3.742 | **<0.001** |
| **Stroke and Aphasia Quality of Life Scale (SAQOL-39)** | | | | |
| Physical score | 1.82  (1.29,2.71) | 2.88±1.07 | -4.542 | **<0.001** |
| Communication score | 1.88±0.63 | 2.80±0.83 | -4.296 | **<0.001** |
| Psychosocial scores | 2.00  (1.50,2.64) | 2.96±0.91 | -4.146 | **<0.001** |
| SAQOL-39 total score | 71.00  (59.00,101.00) | 112.81±34.48 | -4.421 | **<0.001** |
| Bold *P* values indicate statistically significant between-group differences (*P*<0.05). | | | | |

**2.Comparison of language function scores before and after treatment in the SA group**

Following the intervention, the SA group demonstrated significant improvements across all measured language-related outcomes. Within-group analyses showed that, compared with baseline (T0), post-intervention scores on the WAB were significantly increased for object naming, word fluency, sentence completion, responsive speech, and total naming (all *P* < 0.05).

Similarly, relative to baseline (T0), post-intervention scores on the BNT, including spontaneous naming, selective naming, and prompt naming, were significantly higher (all *P* < 0.05). Correspondingly, BNT misnamed scores were significantly reduced after the intervention (*P* < 0.05), indicating improved naming accuracy.

Compared with baseline (T0), scores on the SAQOL-39, including the physical, communication, and psychosocial scores, as well as the SAQOL-39 total score, were all significantly improved after the intervention (all *P* < 0.05) (Table S4).

| **Supplementary Table S4 Within-group comparison of language function in the SA group pre- and post-intervention** | | | | |
| --- | --- | --- | --- | --- |
| Item | SA group (n = 30) | | t/Z | *P* |
|  | T0 (pre) | T1 (post) |  |  |
| **WAB Naming Subtest (WAB)** | | | | |
| Object naming score | 8.00  (3.00,22.25) | 34.00  (18.50,43.00) | -4.784 | **<0.001** |
| Word fluency score | 0.00  (0.00,3.25) | 5.00  (1.75,8.50) | -3.811 | **<0.001** |
| Sentence completion score | 0.00  (0.00,4.50) | 6.00  (2.00,10.00) | -4.242 | **<0.001** |
| Responsive speech score | 0.00  (0.00,6.00) | 8.00  (2.00,10.00) | -4.224 | **<0.001** |
| WAB naming total score | 9.00  (4.50,33.00) | 50.00  (30.75,67.75) | -4.783 | **<0.001** |
| **Boston Naming Test (BNT)** | | | | |
| Spontaneous naming score | 3.00  (1.00,6.25) | 6.50  (4.00,13.50) | -4.719 | **<0.001** |
| Selective naming score | 4.50  (0.75,5.00) | 5.00  (2.00,8.25) | -2.245 | **0.023** |
| Prompt naming score | 4.00  (2.00,5.50) | 5.50  (2.75,7.25) | -1.971 | **0.048** |
| Misnamed score | 15.47±7.79 | 9.50  (4.00,16.00) | -4.547 | **<0.001** |
| **Stroke and Aphasia Quality of Life Scale (SAQOL-39)** | | | | |
| Physical score | 2.00  (1.59,2.59) | 3.35±1.02 | -4.176 | **<0.001** |
| Communication score | 1.92±0.66 | 3.20±0.84 | -4.714 | **<0.001** |
| Psychosocial scores | 1.93  (1.50,2.93) | 3.57±0.93 | -4.765 | **<0.001** |
| SAQOL-39 total score | 83.00  (61.00,109.00) | 132.47±34.27 | -4.66 | **<0.001** |
| Bold *P* values indicate statistically significant between-group differences (*P*<0.05). | | | | |

**3.Between-group comparison of pre-intervention tract diffusivity between the SA group and the control group**

Compared with the control group, the SA group showed no statistically significant differences in axial diffusivity (AD), radial diffusivity (RD), and mean diffusivity (MD) across all examined white matter tracts (all *P* > 0.05).

However, significant between-group differences were observed in fractional anisotropy (FA) values for the left inferior fronto-occipital fasciculus (left IFOF), left inferior longitudinal fasciculus (left ILF), and left uncinate fasciculus (left UF) (all *P* < 0.05), whereas no statistically significant differences were found in the FA values of the remaining tracts (all *P* > 0.05) (Table S5).

| **Supplementary Table S5 Between-group comparison of tract diffusivity between the SA group and the control group before intervention** | | | | |
| --- | --- | --- | --- | --- |
| Item | Control group (n=27) | SA group (n=30) | Z | *P* |
| **Axial diffusivity (AD)** | | | | |
| Left_IFOF | 12.810(12.360,13.480)*10^-4^ | 13.150(12.763,13.500)*10^-4^ | -1.103 | 0.274 |
| Right_IFOF | 12.930(12.670,13.190)*10^-4^ | 12.730(12.568,13.150)*10^-4^ | -0.727 | 0.472 |
| Left_ILF | 12.910(12.500,13.990)*10^-4^ | 12.955(12.772,13.145)*10^-4^ | -0.184 | 0.858 |
| Right_ILF | 12.270(11.810,12.630)*10^-4^ | 12.425(12.130,12.765)*10^-4^ | -1.199 | 0.234 |
| Left_SLF | 11.710(11.270,12.400)*10^-4^ | 11.610(11.143,12.600)*10^-4^ | -0.096 | 0.927 |
| Right_SLF | 11.430(11.120,11.750)*10^-4^ | 11.340(10.940,11.575)*10^-4^ | -1.455 | 0.148 |
| Left_UF | 12.210(11.630,12.650)*10^-4^ | 12.270(11.980,12.760)*10^-4^ | -0.951 | 0.346 |
| Right_UF | 12.420(12.090,12.810)*10^-4^ | 12.405(12.148,12.708)*10^-4^ | -0.024 | 0.984 |
| **Radial diffusivity (RD)** | | | | |
| Left_IFOF | 0.725(0.652,0.837)*10^-3^ | 0.689(0.610,0.763)*10^-3^ | -1.502 | 0.135 |
| Right_IFOF | 0.616(0.594,0.639)*10^-3^ | 0.609(0.569,0.649)*10^-3^ | -0.560 | 0.581 |
| Left_ILF | 0.686(0.650,0.813)*10^-3^ | 0.650(0.620,0.730)*10^-3^ | -1.798 | 0.073 |
| Right_ILF | 0.623(0.605,0.658)*10^-3^ | 0.626(0.585,0.655)*10^-3^ | -0.288 | 0.778 |
| Left_SLF | 0.688(0.623,0.786)*10^-3^ | 0.690(0.623,0.774)*10^-3^ | -0.264 | 0.797 |
| Right_SLF | 0.601(0.566,0.659)*10^-3^ | 0.578(0.553,0.611)*10^-3^ | -1.527 | 0.129 |
| Left_UF | 0.740(0.605,0.858)*10^-3^ | 0.675(0.592,0.785)*10^-3^ | -1.550 | 0.123 |
| Right_UF | 0.519(0.501,0.554)*10^-3^ | 0.530(0.496,0.562)*10^-3^ | -0.272 | 0.791 |
| **Mean diffusivity (MD)** | | | | |
| Left_IFOF | 0.897(0.847,1.007)*10^-3^ | 0.895(0.843,0.950)*10^-3^ | -0.663 | 0.513 |
| Right_IFOF | 0.842(0.806,0.867)*10^-3^ | 0.831(0.804,0.870)*10^-3^ | -0.511 | 0.614 |
| Left_ILF | 0.891(0.861,0.985)*10^-3^ | 0.866(0.844,0.925)*10^-3^ | -1.519 | 0.131 |
| Right_ILF | 0.830(0.802,0.848)*10^-3^ | 0.831(0.797,0.861)*10^-3^ | -0.232 | 0.821 |
| Left_SLF | 0.863(0.790,0.965)*10^-3^ | 0.846(0.779,0.956)*10^-3^ | -0.312 | 0.760 |
| Right_SLF | 0.781(0.758,0.815)*10^-3^ | 0.764(0.729,0.793)*10^-3^ | -1.583 | 0.115 |
| Left_UF | 0.900(0.790,0.978)*10^-3^ | 0.859(0.795,0.934)*10^-3^ | -0.895 | 0.376 |
| Right_UF | 0.761(0.746,0.786)*10^-3^ | 0.765(0.746,0.778)*10^-3^ | -0.36 | 0.724 |
| **Fractional anisotropy (FA)** | | | | |
| Left_IFOF | 0.353(0.312,0.391) | 0.398(0.355,0.451) | -2.669 | **0.007** |
| Right_IFOF | 0.442(0.412,0.461) | 0.449(0.422,0.472) | -0.751 | 0.460 |
| Left_ILF | 0.383(0.316,0.414) | 0.411(0.374,0.440) | -2.381 | **0.017** |
| Right_ILF | 0.413(0.396,0.433) | 0.424(0.396,0.446) | -1.327 | 0.189 |
| Left_SLF | 0.312(0.281,0.367) | 0.334(0.306,0.377) | -1.295 | 0.200 |
| Right_SLF | 0.417(0.363,0.432) | 0.416(0.390,0.437) | -0.607 | 0.552 |
| Left_UF | 0.291(0.280,0.374) | 0.382(0.289,0.442) | -2.509 | **0.012** |
| Right_UF | 0.501(0.469,0.524) | 0.496(0.457,0.528) | -0.176 | 0.868 |
| Bold *P* values indicate statistically significant between-group differences (*P*<0.05). | | | | |

**4. Between-group comparison of pre-intervention topological properties between the SA group and the control group**

Compared with the control group, the SA group showed a statistically significant difference only in the clustering coefficient among the small-world properties (*P* < 0.05), whereas no significant between-group differences were observed for the other small-world properties (all *P* > 0.05).

Regarding network efficiency, the SA group exhibited a statistically significant difference in local efficiency compared with the control group (*P* < 0.05), whereas no significant between-group difference was found in global efficiency (*P* > 0.05) (Table S6).

| **Supplementary Table S6 Between-group comparison of topological properties between the SA group and the control group before intervention** | | | | |
| --- | --- | --- | --- | --- |
| Item | Control group  (n = 27) | SA group  (n = 30) | Z | *P* |
| **Small-world properties** | | | | |
| Small-worldness | 0.386(0.363,0.406) | 0.384(0.366,0.402) | -0.200 | 0.846 |
| Normalized clustering coefficient | 0.388(0.382,0.411) | 0.389(0.374,0.413) | -0.615 | 0.544 |
| Normalized characteristic path length | 0.354(0.352,0.361) | 0.354(0.352,0.357) | -0.448 | 0.660 |
| Clustering coefficient | 0.023(0.021,0.025) | 0.022(0.020,0.024) | -2.063 | **0.039** |
| Characteristic path length | 52.136(46.953,60.928) | 51.286(47.554,54.924) | -0.799 | 0.429 |
| **Network efficiency** | | | | |
| Global efficiency | 0.002(0.002,0.003) | 0.002(0.002,0.003) | -0.652 | 0.520 |
| Local efficiency | 0.028(0.027,0.029) | 0.026(0.024,0.028) | -2.981 | **0.002** |
| Bold *P* values indicate statistically significant between-group differences (*P*<0.05). | | | | |

**5. Intergroup comparison of tract diffusivity differences in the ventral-dorsal structural brain network**

For axial diffusivity (AD), compared with the control group, the SA group showed statistically significant differences in the change values (T1-T0) for the left inferior fronto-occipital fasciculus (left IFOF), left inferior longitudinal fasciculus (left ILF), left superior longitudinal fasciculus (left SLF), and left uncinate fasciculus (left UF) (all *P* < 0.00625 after Bonferroni correction), No other between-group differences survived Bonferroni correction.

For radial diffusivity (RD), compared with the control group, the SA group demonstrated statistically significant differences in the change values (T1-T0) for the left IFOF, left ILF, and left SLF (all *P* < 0.00625 after Bonferroni correction).The remaining tracts diffusivity did not show statistically significant differences (*P* > 0.05).

For mean diffusivity (MD), the SA group exhibited statistically significant differences from the control group in the change values (T1-T0) for the left IFOF (*P* < 0.001), left ILF (*P* = 0.001), and left SLF (*P* < 0.001). No other between-group differences survived Bonferroni correction. For fractional anisotropy (FA), no statistically significant between-group differences were observed in the change values (T1-T0) for any of the examined tracts (*P* > 0.05) (Table S7).

| **Supplementary Table S7 Intergroup comparison of the differences in tract diffusivity of pre (T0)/post (T1) rehabilitation training** | | | | | | | | |
| --- | --- | --- | --- | --- | --- | --- | --- | --- |
| Item | Control group (n = 27) | | | SA group (n = 30) | | | Z/F | *P* |
|  | T0 (pre) | T1 (post) | T1–T0 | T0 (pre) | T1 (post) | T1–T0 |  |  |
| **Axial diffusivity (AD)** | | | | | | | | |
| Left_IFOF | 12.810  (12.360,13.480)*10^-4^ | 13.410  (12.700,13.990)*10^-4^ | 38.00  (29.00,51.00)*10^-6^ | 13.150  (12.763,13.500)*10^-4^ | 13.085  (12.710,13.450)*10^-4^ | -4.50  (-16.75,5.25)*10^-6^ | -6.283 | **<0.001** |
| Right_IFOF | 12.930  (12.670,13.190)*10^-4^ | 12.900  (12.630,13.160)*10^-4^ | 2.00  (-11.00,9.00)*10^-6^ | 12.730  (12.568,13.150)*10^-4^ | 12.675  (12.518,12.962)*10^-4^ | -8.50  (-24.50,5.25)*10^-6^ | -2.039 | 0.041 |
| Left_ILF | 12.910  (12.500,13.990)*10^-4^ | 13.200  (12.760,13.820)*10^-4^ | 9.00  (0.01,30.00)*10^-6^ | 12.955  (12.772,13.145)*10^-4^ | 12.940  (12.440,13.223)*10^-4^ | -10.50  (-22.25,8.00)*10^-6^ | -3.230 | **0.001** |
| Right_ILF | 12.270  (11.810,12.630)*10^-4^ | 12.310  (11.960,12.680)*10^-4^ | (5.26±20.66)*10^-6^ | 12.425  (12.130,12.765)*10^-4^ | 12.335  (12.000,12.745)*10^-4^ | (-5.53±20.19)*10^-6^ | -1.993 | 0.051 |
| Left_SLF | 11.710  (11.270,12.400)*10^-4^ | 11.880  (11.360,12.640)*10^-4^ | 20.00  (-3.00,46.00)*10^-6^ | 11.610  (11.143,12.600)*10^-4^ | 11.420  (11.113,12.468)*10^-4^ | -8.00  (-34.25,19.25)*10^-6^ | -3.005 | **0.002** |
| Right_SLF | 11.430  (11.120,11.750)*10^-4^ | 11.430  (11.120,11.800)*10^-4^ | 8.00  (-6.00,18.00)*10^-6^ | 11.340  (10.940,11.575)*10^-4^ | 11.180  (10.943,11.623)*10^-4^ | -2.50  (-19.50,11.00)*10^-6^ | -1.935 | 0.053 |
| Left_UF | 12.210  (11.630,12.650)*10^-4^ | 12.490  (11.710,13.320)*10^-4^ | 34.00  (-2.00,68.00)*10^-6^ | 12.270  (11.980,12.760)*10^-4^ | 12.245  (11.818,12.780)*10^-4^ | -2.00  (-17.25,10.75)*10^-6^ | -2.893 | **0.003** |
| Right_UF | 12.420  (12.090,12.810)*10^-4^ | 12.320  (11.910,12.690)*10^-4^ | -3.00  (-23.00,15.00)*10^-6^ | 12.405  (12.148,12.708)*10^-4^ | 12.315  (12.048,12.608)*10^-4^ | -7.50  (-29.25,6.50)*10^-6^ | -1.119 | 0.267 |
| **Radial diffusivity (RD)** | | | | | | | | |
| Left_IFOF | 0.725  (0.652,0.837)*10^-3^ | 0.760  (0.697,0.882)*10^-3^ | 34.00  (21.00,51.00)*10^-6^ | 0.689  (0.610,0.763)*10^-3^ | 0.695  (0.618,0.765)*10^-3^ | -0.50  (-14.50,10.00)*10^-6^ | -4.932 | **<0.001** |
| Right_IFOF | 0.616  (0.594,0.639)*10^-3^ | 0.623  (0.593,0.647)*10^-3^ | 1.00  (-13.00,15.00)*10^-6^ | 0.609  (0.569,0.649)*10^-3^ | 0.609  (0.569,0.640)*10^-3^ | -0.50  (-8.75,7.25)*10^-6^ | -0.672 | 0.507 |
| Left_ILF | 0.686  (0.650,0.813)*10^-3^ | 0.696  (0.647,0.848)*10^-3^ | 10.00  (-1.00,22.00)*10^-6^ | 0.650  (0.620,0.730)*10^-3^ | 0.654  (0.615,0.716)*10^-3^ | -2.00  (-11.00,10.00)*10^-6^ | -2.710 | **0.006** |
| Right_ILF | 0.623  (0.605,0.658)*10^-3^ | 0.629  (0.608,0.656)*10^-3^ | -2.00  (-15.00,10.00)*10^-6^ | 0.626  (0.585,0.655)*10^-3^ | 0.624  (0.588,0.648)*10^-3^ | 0.01  (-8.00,8.75)*10^-6^ | -0.056 | 0.959 |
| Left_SLF | 0.688  (0.623,0.786)*10^-3^ | 0.715  (0.638,0.822)*10^-3^ | 21.00  (5.00,52.00)*10^-6^ | 0.690  (0.623,0.774)*10^-3^ | 0.704  (0.622,0.762)*10^-3^ | 0.50  (-13.50,12.50)*10^-6^ | -3.405 | **<0.001** |
| Right_SLF | 0.601  (0.566,0.659)*10^-3^ | 0.600  (0.571,0.650)*10^-3^ | 2.00  (-8.00,10.00)*10^-6^ | 0.578  (0.553,0.611)*10^-3^ | 0.581  (0.552,0.596)*10^-3^ | 0.50  (-8.50,10.50)*10^-6^ | -0.624 | 0.538 |
| Left_UF | 0.740  (0.605,0.858)*10^-3^ | 0.754  (0.649,0.890)*10^-3^ | 22.00  (-17.00,72.00)*10^-6^ | 0.675  (0.592,0.785)*10^-3^ | 0.664  (0.585,0.790)*10^-3^ | 2.00  (-12.25,12.00)*10^-6^ | -1.679 | 0.094 |
| Right_UF | 0.519  (0.501,0.554)*10^-3^ | 0.521  (0.504,0.545)*10^-3^ | -2.00  (-20.00,17.00)*10^-6^ | 0.530  (0.496,0.562)*10^-3^ | 0.528  (0.495,0.567)*10^-3^ | 1.00  (-8.25,10.25)*10^-6^ | -0.016 | 0.991 |
| **Mean diffusivity (MD)** | | | | | | | | |
| Left_IFOF | 0.897  (0.847,1.007)*10^-3^ | 0.935  (0.889,1.054)*10^-3^ | 35.00  (24.00,56.00)*10^-6^ | 0.895  (0.843,0.950)*10^-3^ | 0.896  (0.840,0.953)*10^-3^ | -1.50  (-13.75,6.50)*10^-6^ | -5.611 | **<0.001** |
| Right_IFOF | 0.842  (0.806,0.867)*10^-3^ | 0.844  (0.819,0.878)*10^-3^ | -1.00  (-6.00,11.00)*10^-6^ | 0.831  (0.804,0.870)*10^-3^ | 0.827  (0.802,0.860)*10^-3^ | -3.50  (-9.75,3.25)*10^-6^ | -1.295 | 0.198 |
| Left_ILF | 0.891  (0.861,0.985)*10^-3^ | 0.896  (0.863,1.024)*10^-3^ | 9.00  (-2.00,24.00)*10^-6^ | 0.866  (0.844,0.925)*10^-3^ | 0.865  (0.843,0.912)*10^-3^ | -2.00  (-15.25,4.75)*10^-6^ | -3.118 | **0.001** |
| Right_ILF | 0.830  (0.802,0.848)*10^-3^ | 0.828  (0.814,0.846)*10^-3^ | 2.00  (-11.00,10.00)*10^-6^ | 0.831  (0.797,0.861)*10^-3^ | 0.826  (0.806,0.856)*10^-3^ | -1.50  (-6.25,6.25)*10^-6^ | -0.984 | 0.330 |
| Left_SLF | 0.863  (0.790,0.965)*10^-3^ | 0.866  (0.806,0.994)*10^-3^ | 24.00  (2.00,47.00)*10^-6^ | 0.846  (0.779,0.956)*10^-3^ | 0.847  (0.785,0.921)*10^-3^ | -2.50  (-17.50,10.25)*10^-6^ | -3.605 | **<0.001** |
| Right_SLF | 0.781  (0.758,0.815)*10^-3^ | 0.777  (0.764,0.812)*10^-3^ | 1.00  (-4.00,10.00)*10^-6^ | 0.764  (0.729,0.793)*10^-3^ | 0.765  (0.738,0.779)*10^-3^ | -1.50  (-12.00,0.85)*10^-6^ | -1.440 | 0.152 |
| Left_UF | 0.900  (0.790,0.978)*10^-3^ | 0.908  (0.819,1.059)*10^-3^ | 22.00  (-10.00,68.00)*10^-6^ | 0.859  (0.795,0.934)*10^-3^ | 0.849  (0.791,0.934)*10^-3^ | -2.50  (-15.00,13.00)*10^-6^ | -2.046 | 0.040 |
| Right_UF | 0.761  (0.746,0.786)*10^-3^ | 0.757  (0.743,0.784)*10^-3^ | -4.00  (-12.00,12.00)*10^-6^ | 0.765  (0.746,0.778)*10^-3^ | 0.760  (0.743,0.780)*10^-3^ | -3.50  (-13.75,8.25)*10^-6^ | -0.432 | 0.671 |
| **Fractional anisotropy (FA)** | | | | | | | | |
| Left_IFOF | 0.353  (0.312,0.391) | 0.348  (0.305,0.394) | -37.36  (-131.21,36.03)*10^-4^ | 0.398  (0.355,0.451) | 0.387  (0.354,0.445) | 0.66  (-76.04,72.36)*10-^4^ | 3.465 | 0.068 |
| Right_IFOF | 0.442  (0.412,0.461) | 0.438  (0.422,0.460) | -32.78  (-156.94,135.67)*10^-4^ | 0.449  (0.422,0.472) | 0.446  (0.419,0.471) | -23.56  (-68.09,21.86)*10^-4^ | -0.112 | 0.918 |
| Left_ILF | 0.383  (0.316,0.414) | 0.380  (0.321,0.412) | -34.40  (-73.71,64.34)*10^-4^ | 0.411  (0.374,0.440) | 0.407  (0.372,0.446) | -2.98  (-64.02,82.15)*10^-4^ | 0.940 | 0.336 |
| Right_ILF | 0.413  (0.396,0.433) | 0.414  (0.388,0.427) | 9.19  (-120.62,132.20)*10^-4^ | 0.424  (0.396,0.446) | 0.425  (0.394,0.445) | -17.47  (-82.83,22.51)*10^-4^ | -0.416 | 0.683 |
| Left_SLF | 0.312  (0.281,0.367) | 0.295  (0.277,0.359) | (-93.30±162.76)*10^-4^ | 0.334  (0.306,0.377) | 0.323  (0.294,0.375) | (-50.39±163.54)*10^-4^ | 0.991 | 0.326 |
| Right_SLF | 0.417  (0.363,0.432) | 0.405  (0.374,0.439) | 18.29  (-93.46,127.80)*10^-4^ | 0.416  (0.390,0.437) | 0.415  (0.398,0.432) | 6.58  (-92.14,50.74)*10^-4^ | -0.655 | 0.520 |
| Left_UF | 0.291  (0.280,0.374) | 0.301  (0.259,0.366) | -4.10  (-118.68,191.32)*10^-4^ | 0.382  (0.289,0.442) | 0.379  (0.293,0.438) | -28.84  (-102.55,59.34)*10^-4^ | 0.013 | 0.910 |
| Right_UF | 0.501  (0.469,0.524) | 0.494  (0.474,0.524) | 8.44  (-134.96,100.96)*10^-4^ | 0.496  (0.457,0.528) | 0.485  (0.449,0.531) | -59.37  (-152.18,88.61)*10^-4^ | -0.671 | 0.510 |
| Bold *P* values indicate statistically significant between-group differences (*P*<0.05).  Z values indicate Mann–Whitney U tests; F values indicate ANCOVA adjusted for the corresponding baseline value. | | | | | | | | |

**6. Between-group comparison of changes in topological properties in the ventral-dorsal structural language network**

In terms of small-world properties, compared with the control group, the SA group showed statistically significant differences in the change scores (T1−T0) for small-worldness, normalized clustering coefficient, and clustering coefficient (all *P* < 0.05), whereas no significant between-group differences were observed in the change scores of the remaining small-world metrics (all *P* > 0.05).

With regard to network efficiency, compared with the control group, the SA group showed a statistically significant difference only in the change score (T1−T0) for local efficiency (*P* < 0.05), whereas no significant between-group difference was found in the change score for global efficiency (*P* > 0.05) (Table S8).

| **Supplementary Table S8 Intergroup comparison of the differences in topological properties of pre (T0)/post (T1) rehabilitation training** | | | | | | | | |
| --- | --- | --- | --- | --- | --- | --- | --- | --- |
| Item | Control group (n = 27) | | | SA group (n = 30) | | | Z/F | *P* |
|  | T0  (pre) | T1  (post) | T1-T0  (post-pre) | T0  (pre) | T1  (post) | T1-T0  (post-pre) |  |  |
| **Small-world properties** | | | | | | | | |
| Small-worldness | 0.386  (0.363,0.406) | 0.393  (0.374,0.453) | 0.0106  (-0.0094,0.0498) | 0.384  (0.366,0.402) | 0.371  (0.354,0.392) | -0.0063  (-0.0295,0.0043) | -2.637 | **0.008** |
| Normalized clustering coefficient | 0.388  (0.382,0.411) | 0.395  (0.376,0.478) | 0.0132  (-0.0069,0.0569) | 0.389  (0.374,0.413) | 0.381  (0.366,0.405) | -0.0101  (-0.0276,0.0078) | -2.381 | **0.017** |
| Normalized characteristic path length | 0.354  (0.352,0.361) | 0.353  (0.351,0.358) | -0.0010  (-0.0034,0.0041) | 0.354  (0.352,0.357) | 0.354  (0.351,0.359) | -0.0001  (-0.0037,0.0039) | -0.543 | 0.595 |
| Clustering coefficient | 0.023  (0.021,0.025) | 0.023  (0.021,0.024) | 0.0001  (-0.0024,0.0007) | 0.022  (0.020,0.024) | 0.023  (0.021,0.024) | 0.0013  (-0.0001,0.0022) | 6.859 | **0.011** |
| Characteristic path length | 52.136  (46.953,60.928) | 53.133  (48.693,61.342) | 1.8337  (-1.1600,6.1448) | 51.286  (47.554,54.924) | 50.234  (47.942,55.012) | -0.3700  (-2.6708,2.9646) | -1.287 | 0.201 |
| **Network efficiency** | | | | | | | | |
| Global efficiency | 0.002  (0.002,0.003) | 0.002  (0.002,0.003) | -0.00090  (-0.00023,0.00005) | 0.0024  (0.002,0.003) | 0.002  (0.002,0.003) | 0.00001  (-0.00013,0.00012) | -1.135 | 0.260 |
| Local efficiency | 0.028  (0.027,0.029) | 0.027  (0.025,0.028) | -0.00034  (-0.00205,0.00048) | 0.026  (0.024,0.028) | 0.027  (0.025,0.028) | 0.00136  (0.00019,0.00247) | 13.332 | **<0.001** |
| Bold *P* values indicate statistically significant between-group differences (*P*<0.05).  Z values indicate Mann–Whitney U tests; F values indicate ANCOVA adjusted for the corresponding baseline value. | | | | | | | | |

**7. Exploratory hierarchical regression analyses of naming improvement**

Among 57 participants with complete clinical and neuroimaging data, Model 1, including treatment assignment and clinical covariates, explained 26.9% of the variance in change in WAB naming total score (adjusted R² = 0.148; F (8, 48) = 2.211, *P* = 0.043). Assignment to the scalp acupuncture group was associated with greater improvement in WAB naming total score (B = 12.931, standardized β = 0.378, *P* = 0.005).

After adding changes in left inferior fronto-occipital fasciculus axial diffusivity, left uncinate fasciculus axial diffusivity, and small-worldness, the explained variance increased from 0.269 to 0.292. However, this increment was not statistically significant (ΔR² = 0.023, F-change [3,45] = 0.487, *P* = 0.693). None of the imaging change measures was independently associated with naming improvement in the expanded model.

In an influence analysis excluding six observations with Cook’s distance >4/n (0.070), the incremental contribution of imaging changes remained non-significant (ΔR² = 0.051, F-change [3,39] = 0.982, *P* = 0.411). Bias-corrected and accelerated bootstrap 95% confidence intervals based on 5,000 resamples included zero for all three imaging change measures (Table S9).

**Supplementary Table S9 Sensitivity analyses for exploratory regression models of change in WAB naming total score**

**Panel A. Model performance**

| Analysis/model | n | R² | Adjusted R² | ΔR² | F-change | *P* for F-change |
| --- | --- | --- | --- | --- | --- | --- |
| **Primary analysis: Model 1 (treatment and clinical covariates)** | 57 | 0.269 | 0.148 | — | — | — |
| **Primary analysis: Model 2** (Model 1 + imaging changes) | 57 | 0.292 | 0.119 | 0.023 | 0.487 | 0.693 |
| **Sensitivity analysis excluding Cook’s D >0.070: Model 1** | 51 | 0.269 | 0.130 | — | — | — |
| **Sensitivity analysis excluding Cook’s D >0.070: Model 2** | 51 | 0.320 | 0.129 | 0.051 | 0.982 | 0.411 |

**Panel B. Regression estimates**

| **Predictor** | **Primary analysis, B (standardized β), *P*** | **Excluding Cook’s D >0.070, B (standardized β), *P*** | **BCa bootstrap 95% CI for B*** |
| --- | --- | --- | --- |
| **Model 1** |  |  |  |
| **SA group** | **12.931 (0.378), 0.005** | **12.349 (0.464), 0.002** | **3.591** - **23.354** |
| **Model 2** |  |  |  |
| **SA group** | **9.227 (0.270), 0.187** | **5.275 (0.198), 0.363** | **−5.493** - **27.623** |
| **Left IFOF AD change (×10⁻⁶ mm²/s)** | −0.0339 (−0.081), 0.766 | −0.1287 (−0.408), 0.211 | −0.2943 - 0.2048 |
| **Left UF AD change (×10⁻⁶ mm²/s)** | −0.0188 (−0.089), 0.665 | 0.0170 (0.105), 0.662 | −0.1186 - 0.1449 |
| **Small-worldness change** | **−20.676 (−0.060), 0.680** | **−17.102 (−0.062), 0.682** | **−144.706 - 56.343** |

**Note:** Model 1 included treatment group, baseline WAB naming total score, age, sex, education years, disease duration, stroke type, and aphasia type. Model 2 additionally included theoretically selected changes in left inferior fronto-occipital fasciculus (IFOF) axial diffusivity, left uncinate fasciculus (UF) axial diffusivity, and small-worldness. The Cook’s D sensitivity analysis excluded six observations with Cook’s distance >0.070. BCa bootstrap confidence intervals were based on 5,000 resamples; 3,975 valid bootstrap samples contributed to coefficient-level interval estimation. Standardized β values derive from ordinary least-squares regression; bootstrap intervals refer to unstandardized B coefficients.

**References**

[1]	HICKOK G, POEPPEL D. Dorsal and ventral streams: a framework for understanding aspects of the functional anatomy of language[J]. Cognition, 2004,92(1-2): 67-99.

[2]	HICKOK G, POEPPEL D. The cortical organization of speech processing[J]. Nat Rev Neurosci, 2007,8(5): 393-402.

[3]	MORI S, OISHI K, JIANG H, et al. Stereotaxic white matter atlas based on diffusion tensor imaging in an ICBM template[J]. Neuroimage, 2008,40(2): 570-582.

[4]	GONG G, HE Y, CONCHA L, et al. Mapping anatomical connectivity patterns of human cerebral cortex using in vivo diffusion tensor imaging tractography[J]. Cereb Cortex, 2009,19(3): 524-536.

[5]	CUI Z, ZHONG S, XU P, et al. PANDA: a pipeline toolbox for analyzing brain diffusion images[J]. Front Hum Neurosci, 2013,7: 42.

[6]	FARIA A V, JOEL S E, ZHANG Y, et al. Atlas-based analysis of resting-state functional connectivity: evaluation for reproducibility and multi-modal anatomy-function correlation studies[J]. Neuroimage, 2012,61(3): 613-621.

[7]	WANG J, WANG X, XIA M, et al. GRETNA: a graph theoretical network analysis toolbox for imaging connectomics[J]. Front Hum Neurosci, 2015,9: 386.

[8]	CHEN X, CHEN L, ZHENG S, et al. Disrupted Brain Connectivity Networks in Aphasia Revealed by Resting-State fMRI[J]. Front Aging Neurosci, 2021,13: 666301.
